# Supplementary material for: Development of chemokine network inhibitors using combinatorial saturation mutagenesis
Source: Commun Biol. 2025 Apr 3;8:549. doi: 10.1038/s42003-025-07778-6 (PMC11969024; doi:10.1038/s42003-025-07778-6)
Supplement: Supplementary file 4 — Supplementary Data 2 [file 42003_2025_7778_MOESM4_ESM.zip › SupplementaryData3/scripts/xattune_ic50.tar!/./script/analysis/JK/JK_R30_E66_INS_HD2_THP1_plot.pdf]

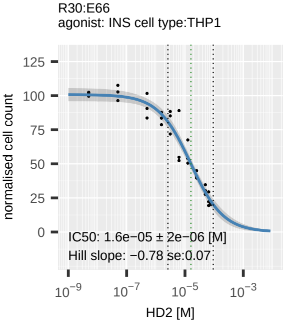

R30:E66
agonist: INS cell type:THP1
125
100
75
50
25
0
normalised cell count
IC50: 1.6e−05 ± 2e−06 [M]
Hill slope: −0.78 se:0.07
10^{-9}
10^{-7}
10^{-5}
10^{-3}
HD2 [M]
